# Donor-derived cell-free DNA in chronic lung allograft dysfunction phenotypes: a pilot study

**DOI:** 10.3389/frtra.2024.1513101

**Published:** 2024-12-23

**Authors:** H. Beeckmans, A. Pagliazzi, P. Kerckhof, R. Hofkens, F. Debackere, A. Zajacova, S. Bos, B. M. Vanaudenaerde, H. de Loor, M. Naesens, R. Vos

**Affiliations:** ^1^ Laboratory of Respiratory Diseases and Thoracic Surgery (BREATHE), Department of CHROMETA, KU Leuven, Leuven, Belgium; ^2^ Nephrology and Renal Transplantation Research Group, Department of Microbiology, Immunology and Transplantation, KU Leuven, Leuven, Belgium; ^3^ Department of Internal Medicine, University Hospitals Leuven, Leuven, Belgium; ^4^ Department of Respiratory Diseases, University Hospitals Leuven, Leuven, Belgium; ^5^ Department of Nephrology and Kidney Transplantation, University Hospitals Leuven, Leuven, Belgium

**Keywords:** lung transplantation, CLAD, dd-cfDNA, rejection, biomarker

## Abstract

Long-term survival after lung transplantation is limited due to chronic lung allograft dysfunction (CLAD), which encompasses two main phenotypes: bronchiolitis obliterans syndrome (BOS) and restrictive allograft syndrome (RAS). Donor-derived cell-free DNA (dd-cfDNA) is a biomarker for (sub)clinical allograft injury and could be a tool for monitoring of lung allograft health across the (pre)clinical spectrum of CLAD. In this proof-of-concept study, we therefore assessed post-transplant plasma dd-cfDNA levels in 20 CLAD patients (11 BOS and 9 RAS) at three consecutive time points free from concurrent infection or acute rejection, during stable condition, preclinical CLAD, and established CLAD (*n* = 3 × 20 samples). Elevated dd-cfDNA levels were detected in 47% of stable samples, in 66% of preclinical CLAD samples, and in 71% of CLAD samples, indicating ongoing allograft injury. However, dd-cfDNA levels exhibited high intra- and interpatient variability and did not significantly differ between BOS and RAS (*p* = 0.25), although the range of dd-cfDNA was higher in RAS. Dd-cfDNA detects ongoing allograft injury in patients with CLAD, which warrants further investigation to improve early detection of CLAD.

## 1 Introduction

Lung transplantation is a life-saving therapeutic option for well-selected patients with end-stage pulmonary diseases. However, long-term outcomes following lung transplantation are significantly hampered by chronic lung allograft dysfunction (CLAD), the major cause of post-transplant mortality. CLAD is characterized by an irreversible decline in pulmonary function, not explained by other causes such as infection or acute rejection, and is defined as a decline in forced expiratory volume in one second (FEV1) of ≥20% from baseline (1). There are two main phenotypes of CLAD: bronchiolitis obliterans syndrome (BOS) and restrictive allograft syndrome (RAS). BOS is characterized by FEV1 decline of ≥20% from baseline with an obstructive pulmonary function test (PFT) (airway obstruction related to small airways disease), in absence of persistent radiologic opacities or total lung capacity (TLC) decline. RAS is defined as a FEV1 decline of ≥20% from baseline accompanied with a restrictive PFT pattern (TLC decline of ≥10% compared to baseline) and persistent opacities on chest x-ray or computed tomography (CT) due to interstitial and/or pleural fibrosis (1). In a mixed phenotype, an obstructive-restrictive PFT and persistent opacities are present.Allograft injury may occur long before it is detectable by conventional diagnostic tools such as FEV1 (2). As a result, early stages of allograft dysfunction may become apparent by changes in small airway function or lung structure, which are evident through other diagnostic modalities, despite stable FEV1 levels (preclinical CLAD).Preclinical BOS is characterized by small airways disease with FEV1 between 80% and 100% of baseline. Patients might display heterogeneous, multi-lobar air trapping on expiratory chest CT and a significant decline in forced expiratory flow at 25% to 75% of forced vital capacity (FEF25-75), a sensitive marker of airflow in small airways. These changes often precede a noticeable decline in FEV1. For example, a patient may show a gradual decline in FEF25-75 and evidence of air trapping on chest CT long before a formal diagnosis of BOS is made based on traditional FEV1 criteria (2–4). In the previous BOS diagnosis ISHLT consensus paper from 2001, BOS stage 0p (potential BOS) was proposed, defined as a persistent decrease in FEV1 of 10%–19% and/or FEF25-75 decline of 25% or more compared to baseline (4). Preclinical RAS, on the other hand, is characterized by persistent CT changes with FEV1 between 80% and 100% of baseline and TLC between 90% and 100% of baseline. Patients may demonstrate chronic interstitial changes on CT, such as reticulations and parenchymal opacities, with or without pleural involvement, without significant decline in FEV1 or TLC. These structural abnormalities, although not meeting the current diagnostic criteria for RAS, suggest ongoing remodeling of the alveoli, pleura, and/or interstitial space, which may eventually progress to clinically overt RAS (2).

Standard allograft monitoring typically includes regular clinical assessments, PFTs, imaging studies such as chest x-rays or CT scans, and periodic bronchoscopy with transbronchial biopsies and bronchoalveolar lavage, which is essential for diagnosing acute rejection, infection, and other post-transplant complications. However, invasive sampling with biopsies, for instance, carries risks such as bleeding and pneumothorax and is prone to sampling errors (5, 6). Similarly, PFTs may be influenced by confounding factors, such as obesity, diaphragm dysfunction, patient effort, etc., which may obscure adequate detection of changes in allograft function. Given these limitations, there is a need for more sensitive and preferably non-invasive biomarkers to monitor lung allograft health.

One promising biomarker is donor-derived cell-free DNA (dd-cfDNA), which is shed into the recipient's bloodstream from injured allograft cells. Dd-cfDNA levels (fraction of genomic DNA levels,%) have been shown to rise before clinical manifestations of allograft rejection, providing a potentially useful tool for early detection of allograft injury (5, 6). Dd-cfDNA has demonstrated good sensitivity for graft injury but lacks specificity for the cause, with elevated levels also seen in infections (7, 8). Several cut-offs have been proposed for allograft injury [ranging from 0.5 to 1% (7–11), also depending on the essay], and for patients with serial dd-cfDNA levels of <1%, fluctuations of ≤70%–73% or less are considered normal (7, 12).

Little is known about dd-cfDNA levels in diverse CLAD phenotypes and during CLAD progression. A pilot study demonstrated elevated% dd-cfDNA at the moment of CLAD diagnosis (during active PFT decline) in 2 patients (% dd-cfDNA around 0.3%–0.4%) (13). Another study (14) demonstrated elevated% dd-cfDNA in established CLAD (median of 1.07% in CLAD samples vs. 0.71% in stable condition), and% dd-cfDNA correlated significantly with FEV1 decline. Two other studies corroborated elevated% dd-cfDNA in CLAD, with levels of 1.6% in CLAD (vs. 0.46% in stable condition) (15) and 2.06% in CLAD (vs. 0.38% in stable condition) (10), respectively.

However, none of these studies made a distinction between BOS and RAS, accurately defined the timepoint of CLAD sampling (i.e., at CLAD diagnosis, after CLAD diagnosis, during active PFT decline), or confounders such as concurrent infections. These are important considerations, since% dd-cfDNA levels may vary depending on time of sampling, method of calculation (16), or the presence of infection. For instance, baseline% dd-cfDNA levels around the time of CLAD diagnosis ± 3 months, with exclusion of samples with concurrent infection, varied between 0.12%–0.47%, depending on the method of calculation (16).

To further explore dd-cfDNA as a biomarker across the CLAD spectrum over time, we conducted a proof-of-concept study, and measured consecutive dd-cfDNA levels in BOS and RAS patients during stable condition, preclinical CLAD and established CLAD. To ensure accurate evaluation of background graft dynamics during progression to CLAD, plasma samples with concurrent infection or rejection were excluded, thereby avoiding confounding events.

## 2 Methods

### 2.1 Sample collection

All lung transplant recipients at our center undergo a surveillance follow-up protocol, which includes clinical evaluation, PFT, blood sampling, chest CT-scan, bronchoscopy with bronchoalveolar lavage, and transbronchial and endobronchial biopsy sampling. Procedures are scheduled at predefined postoperative days (POD 30, 90, 180, 360, 540, and 720), or are performed whenever clinically indicated (e.g., in case of suspected pulmonary infection, acute rejection or CLAD). After POD 360, follow-up visits occur every 3 to 4 months (including spirometry, blood sampling, chest x-ray) and annual CT scan and full PFT (including spirometry, lung volumes, diffusion capacity) is performed in all patients.

Venous blood samples prospectively obtained during procedural follow-up visits are stored into EDTA tubes, plasma is separated by centrifugation at 400_g_ for 10 min at room temperature, and stored in 2 ml aliquots at −80°C until further analysis.

### 2.2 Study population

Our study was approved by the Institutional Ethics Review Board (S66760), and all included patients signed informed consent. We included patients who underwent bilateral lung transplantation between 2010 and 2021 at University Hospitals Leuven. Data of study participants were collected retrospectively. We selected plasma samples from patients diagnosed with BOS and RAS, which were obtained at three consecutive clinical conditions during post-transplant follow-up: during stable condition, preclinical CLAD, and established CLAD. The CLAD status of included patients was evaluated based on the ISHLT 2019 consensus (1).

**Figure 1:**
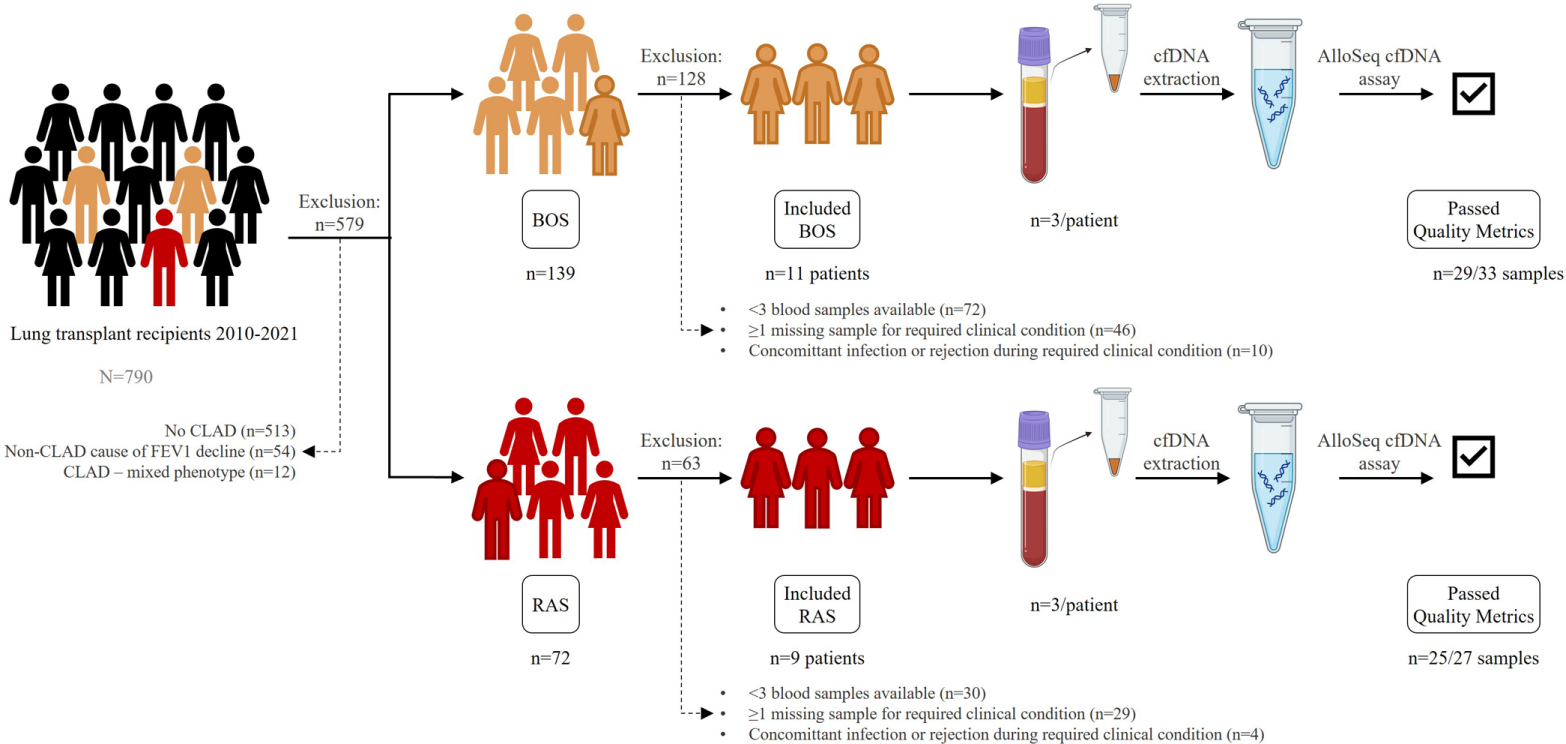
Study set-up. Patients transplanted from 2010 to 2021 were evaluated for inclusion. 11 BOS patients and 9 RAS patients were included. Plasma samples at 3 consecutive clinical conditions were collected, processed for cfDNA extraction, amplified and sequenced with the AlloSeq cfDNA assay. BOS, bronchiolitis obliterans syndrome; cfDNA, cell-free DNA; RAS, restrictive allograft syndrome.

CLAD patients were included based on whether sufficient plasma samples were available, and absence of concomitant infection or rejection at the required clinical condition at sampling (Figure 1). Blood sampling occurred at the following clinical conditions:

(1) Stable allograft function: at baseline allograft function, at least 3 months after transplantation.
(2) Preclinical CLAD, which we defined as:
  • Preclinical BOS: FEV1 decline <20% from baseline, FEF25-75 decline ≥25% from baseline and evidence of air trapping on chest CT.
  • Preclinical RAS: FEV1 decline <20% from baseline and presence of persistent chest CT opacities compatible with RAS (17).
(3) Established CLAD: defined according to the 2019 ISHLT consensus (persistent FEV1 decline ≥20% from baseline) (1):
  • Established BOS: persistent FEV1 decline ≥20% from baseline, with an obstructive PFT pattern, without chest CT opacities.
  • Established RAS: persistent FEV1 decline ≥20% from baseline, with a restrictive PFT pattern (TLC decline of ≥10% compared to baseline) and persistent chest CT opacities.

At each of these time points, concurrent infection or acute rejection was excluded, ensuring that the obtained plasma samples (and dd-cfDNA levels) were not influenced by these confounding events.

### 2.3 Quantification of dd-cfDNA

Cell-free DNA (cfDNA) was extracted from 2 ml thawed plasma samples using the MagMAX™ Cell-Free DNA Isolation Kit (Thermo Scientific, USA). To eliminate possible genomic DNA contamination, size exclusion was performed with Ampure XP Reagent (Beckman Coulter, USA). The entire extraction was performed according the instructions supplied by CareDx. Extracted cfDNA was then amplified using the AlloSeq cfDNA kit (CareDx, USA), which employed multiplex PCR with primers for 202 single nucleotide polymorphisms (SNPs). These SNPs are used to determine the proportion of dd-cfDNA relative to the total cfDNA present in the plasma sample of the recipient. PCR products were subsequently sequenced on a MiSeq system (Illumina, Inc., USA). Sequencing data were analyzed using the CareDx AlloSeq cfDNA software version 2.2.1, obtaining the dd-cfDNA fraction, expressed as a percentage, in the sample. Quality control is based on key metrics such as total reads processed, average marker coverage, and the number of statistical outliers. An overview of the study setup is depicted in Figure 1. A dd-cfDNA level above 0.5% was considered significantly elevated, since all samples were free from infection and acute rejection (18). Also, for dd-cfDNA levels <1%, a serial increase of >70% in dd-cfDNA compared to baseline (value at *stable* condition) was considered significantly elevated.

### 2.4 Statistical analysis

Mixed-effects models with patient as a random effect were used to compare dd-cfDNA levels across different conditions and clinical time points. *P*-values were computed using the Satterthwaite approximation method, and a *p*-value <0.05 was considered significant. Pearson correlation analyses were performed to examine the relationship between dd-cfDNA levels and continuous clinical parameters, such as kidney function and leukocyte counts. Analyses were performed using R version 4.3.2 and GraphPad Prism version 10.

## 3 Results

A total of 20 (11 BOS, 9 RAS) patients were included, with *n* = 3 samples each (*n* = 60 samples). Patient characteristics are depicted in Table 1. After cfDNA extraction, amplification and sequencing, *n* = 54 (90%) samples passed quality metrics. As a result, quantified dd-cfDNA for all 3 clinical timepoints was available for *n* = 9 BOS patients, as 2 BOS patients only had results at the stable condition. In RAS, *n* = 7 patients had results available at all 3 clinical timepoints, since *n* = 1 RAS patient had insufficient cfDNA concentration in stable condition, and *n* = 1 RAS patient had insufficient cfDNA quality at established CLAD. All CLAD (BOS and RAS) patients were included for description of the dd-cfDNA metrics over time. % dd-cfDNA levels in all patients are available in Supplementary Tables S1 and S2.

**Table 1:** Patient characteristics.

	CLAD	BOS	RAS
Number of patients	20	11	9
Age at transplantation, years, median [Q1–Q3]	56 [49–63]	59 [50–63]	52 [48–63]
Native lung disease, *n*			
COPD	15	10	5
ILD	1	0	1
CF	1	0	1
CLAD	2	0	2
BRECT	1	1	0
Sex, female, *n*, %	10, 50%	8, 73%	2, 22%
Type of transplant, *n*, %			
SSLTx	20, 100%	11, 100%	9, 100%
Number of samples passed quality metric/included samples (*n*/*n*)			
Stable condition	19/20	11/11	8/9
Preclinical CLAD	18/20	9/11	9/9
Established CLAD	17/20	9/11	8/9
Time from LTx to stable condition sampling (months), median [Q1–Q3]	6.1 [3.2–12.4]	6 [3.1–6.1]	13 [7.5–18.6]
Timing from LTx to preclinical CLAD sampling (months), median [Q1–Q3]	18.7 [12.4–36.8]	18.2 [12.1–21.7]	36.5 [18.4–55.6]
Timing from LTx to established CLAD sampling (months), median [Q1–Q3]	54.8 [26–74.8]	36.6 [24.5–74.5]	70.1 [50.1–81]

CF, cystic fibrosis; CLAD, chronic lung allograft dysfunction; COPD, chronic obstructive pulmonary disease; BRECT, bronchiectasis; ILD, interstitial lung disease; LTx, lung transplantation; RAS, restrictive allograft syndrome; SSLTx, sequential single lung transplantation.

### 3.1 No CLAD vs. preclinical CLAD vs. established CLAD

**Figure 2:**
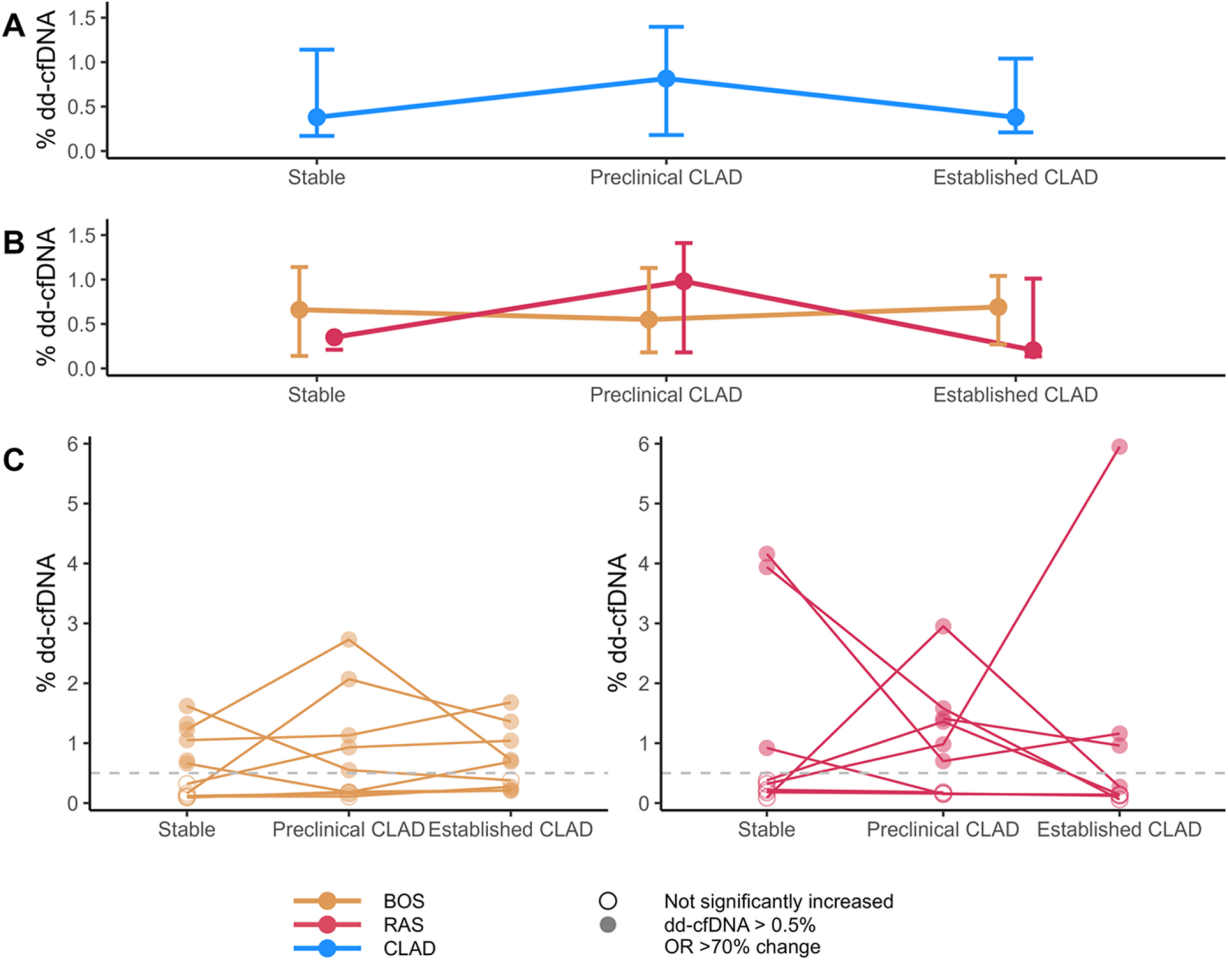
Overview of% dd-cfDNA levels. **(A)** Median and Q1-Q3 of% dd-cfDNA in CLAD samples across the CLAD spectrum. **(B)** Median and Q1-Q3 of% dd-cfDNA in both BOS and RAS across the CLAD spectrum. **(C)** Individual% dd-cfDNA levels over time. The dashed grey line indicates the cut-off value of 0.5% dd-cfDNA. Filled dots indicate% dd-cfDNA values that are either above the cut-off, or (if value < 1%) changed > 70% from the lowest measured value in the patient. BOS, bronchiolitis obliterans syndrome; cfDNA, cell-free DNA; CLAD, chronic lung allograft dysfunction; RAS, restrictive allograft syndrome.

Median% dd-cfDNA levels in CLAD patients, as well as individual values in BOS and RAS patients are depicted in Figure 2. Overall, 47% of stable condition samples demonstrated elevated (>0.5% dd-cfDNA)% dd-cfDNA levels. Moreover, 69% of preclinical and established CLAD samples demonstrated elevated% dd-cfDNA levels (54% > 0.5% dd-cfDNA, 14% < 0.5% dd-cfDNA but with >70% increase from baseline). However, dd-cfDNA levels did not significantly differ between different clinical conditions: preclinical CLAD (median 0.8% dd-cfDNA, 66% of samples elevated) vs. stable (0.4% dd-cfDNA) (*p* = 0.90); established CLAD (median 0.4% dd-cfDNA, 71% of samples elevated) vs. stable (median 0.4% dd-cfDNA) (*p* = 0.95), and individual levels were highly variable. Dd-cfDNA levels were not influenced by concurrent kidney function (*p* = 0.34) or leukocyte count (*p* = 0.99), time since transplant (*p* = 0.76), time to CLAD from sample (*p* = 0.63), time since CLAD diagnosis (*p* = 0.34) or rate of FEV1 decline (*p* = 0.72).

Overall, there was no significant difference in% dd-cfDNA levels between BOS and RAS (*p* = 0.25), although the range of% dd-cfDNA values at each time point was higher in RAS.

### 3.2 BOS

**Figure 3:**
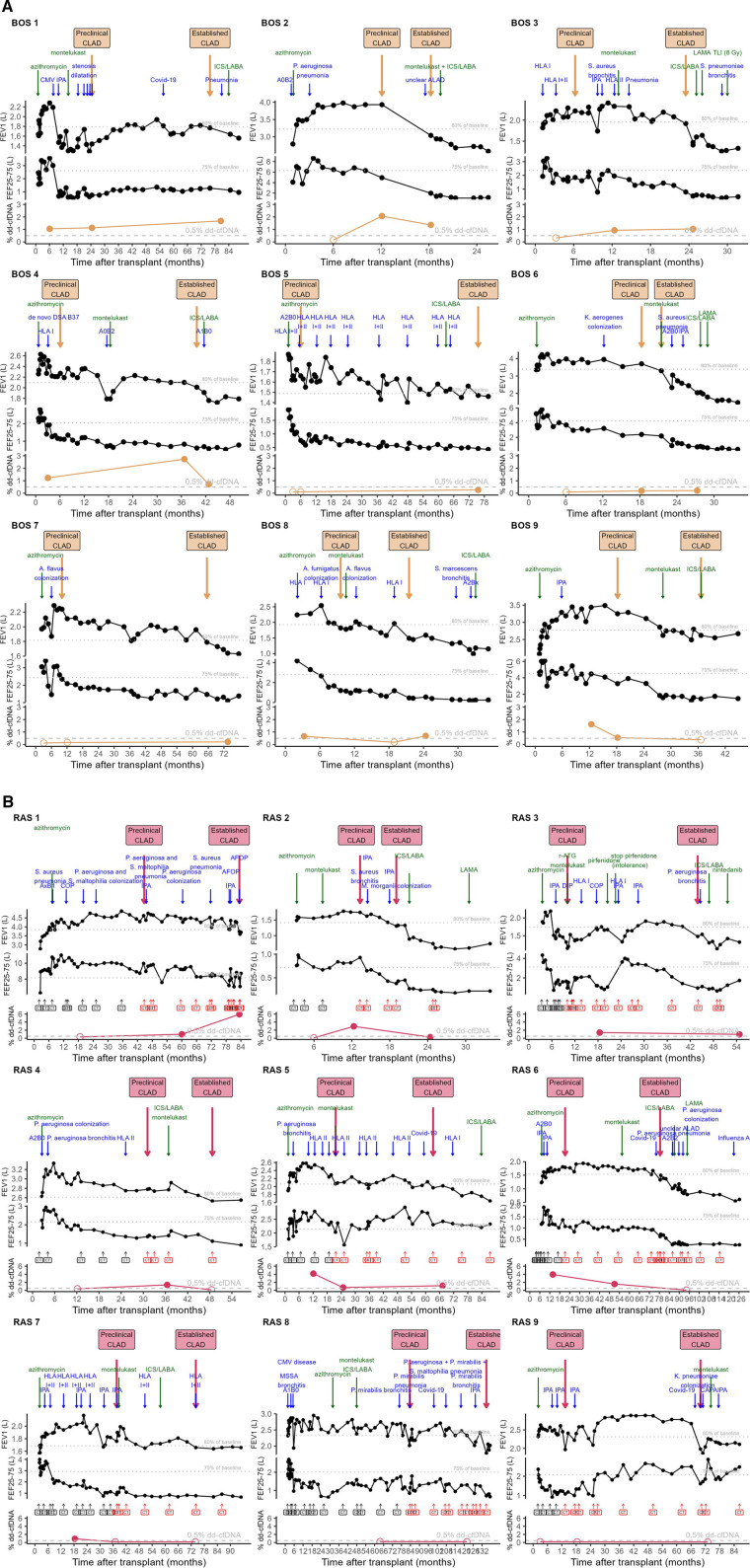
Overview of % dd-cfDNA in relation to the individual patients’ trajectory. **(A)** BOS patients. **(B)** RAS patients. Clinical course of the post-transplant trajectory in individual CLAD patients. Lower graph depicts% dd-cfDNA levels. Upper graph depicts FEV1 in L with 80% of patient baseline (best 2 postoperative values taken at least 3 weeks apart) indicated. Above the FEV1 graph, dates of preclinical CLAD and established CLAD are indicated in yellow arrows for BOS, pink arrows for RAS. Clinical events are indicated in blue arrows, whereas CLAD prevention/treatment start dates are indicated in green arrows. The middle graph depicts FEF25-75 in L with 75% of patient baseline (best 2 postoperative values taken at least 3 weeks apart) indicated. For RAS patients, the moments when a chest CT was performed are indicated, and chest CTs with persistent opacities compatible with RAS are indicated in red. As 2 BOS patients only had results at stable condition, they are not included in this figure (BOS 10 and 11). ACR, acute cellular rejection, indicated with A grade and B grade according to ISHLT classification; AFOP, acute fibrinous and organizing pneumonia; ALAD, acute lung allograft dysfunction without clear cause; CLAD, chronic lung allograft dysfunction; CMV, cytomegalovirus, indicating CMV pneumonitis in BOS patient 1; COP, cryptogenic organizing pneumonia; COVID-19, coronavirus disease 19, caused by SARS-CoV-2; DIP, desquamative interstitial pneumonia; DSA, donor-specific antibodies indicated at date of measurement in the blood; HLA I/II, Human leukocyte antigen antibodies class I/II, indicated with arrows at each moment of measurement in the blood; ICS/LABA, inhaled corticosteroid – long-acting beta2 agonist; IPA, invasive pulmonary aspergillosis, as defined by EORTC/MSGERC and ISHLT consensus; LAMA, long-acting muscarinic antagonist; r-ATG, rabbit-derived anti-thymocyte globulin; TLI, total lymphoid irradiation.

Detailed clinical description of 9 BOS patients with complete results is presented in Figure 3A.

Interestingly, all BOS patients demonstrated elevated% dd-cfDNA levels at either preclinical CLAD and/or established CLAD: *n* = 10 samples > 0.5% dd-cfDNA, *n* = 4 samples <0.5% dd-cfDNA but with >70% increase from stable condition, *n* = 4 samples not significantly elevated dd-cfDNA. Furthermore, 6 out of 11 BOS patients (BOS 1, 4, 8, 9, 10, 11) also had elevated dd-cfDNA levels at stable condition. 4 out of 9 BOS patients (BOS 5-6-7-8) had% dd-cfDNA levels <1% at all clinical timepoints, however with 70% increase in preclinical or established CLAD compared to stable condition.

### 3.3 RAS

Detailed clinical description of RAS patients is depicted in Figure 3B and representative chest CTs are depicted in Supplementary Figure S1.

6 out of 7 RAS patients (RAS 1-2-3-4-5-6) had elevated% dd-cfDNA levels during either preclinical and/or established CLAD. RAS patients 7-8-9 had low levels of % dd-cfDNA during both preclinical and established CLAD moments. In 3 out of 7 RAS patients (RAS 5-6-7)% dd-cfDNA levels were elevated at stable condition.

## 4 Discussion

Our pilot study is the first to examine consecutive dd-cfDNA levels in different clinical conditions over time in CLAD patients, distinguishing between the two main disease phenotypes BOS and RAS. The unique setup, involving longitudinal sampling during the course of allograft remodeling towards CLAD development and methodical exclusion of samples at concurrent acute rejection or infection, allowed us to determine *baseline* dd-cfDNA shedding associated with (ongoing) lung allograft remodeling.

Interestingly, 69% of preclinical and established CLAD samples demonstrated elevated dd-cfDNA, however, with high interindividual heterogeneity in allograft dd-cfDNA dynamics that could not be fully explained by the clinical CLAD spectrum. This variation suggests that CLAD may not progress in a continuous manner, but rather exhibits periods of waxing and waning, with elevated dd-cfDNA levels during periods of ongoing injury, and potentially lower dd-cfDNA shedding between these episodes. Although larger cohort studies are needed to further assess dd-cfDNA levels across the CLAD spectrum and trajectory. There was no significant difference in dd-cfDNA levels between BOS and RAS phenotypes of CLAD, although the range of dd-cfDNA values was higher in RAS patients, as previously demonstrated (19). This higher variability in RAS may reflect the more diffuse and severe nature of tissue injury and remodeling seen in this phenotype, although further studies are needed to confirm this. Interestingly, 47% of stable condition samples exhibited elevated% dd-cfDNA levels. Only one patient (BOS 4) demonstrated accompanying *de novo* DSAs at stable condition. This unexpected finding suggests that subclinical graft injury (i.e., by subclinical antibody-mediated rejection or acute cellular rejection) might be more common than previously thought, even in seemingly stable patients, which requires further study.

There are some limitations to our study: the single-center set-up, the limited sample size and inherent selection bias due to exclusion of samples during concurrent acute rejection or infection. Also, our study used long-term stored, frozen plasma samples obtained from EDTA tubes rather than specialized DNA collection tubes, which may not be standard practice at all centers. Despite this, 90% of our samples yielded results that passed quality metrics. This suggests that secondary site sampling, freezing and prolonged storage are possible, expanding the options for setting up dd-cfDNA measurement in a limited number of (centralized) laboratories to cover several transplant programs.

Our findings highlight the need for a multifaceted approach for monitoring and managing CLAD. While dd-cfDNA shows promise as a biomarker for detecting graft injury, its variability and lack of specificity highlight the importance of integrating it with other diagnostic modalities. Additionally, intrapatient variability makes it difficult to define a clear cut-off for all patients to assess CLAD risk. However, an increase in dd-cfDNA in a specific patient may signal ongoing allograft injury, warranting closer follow-up and investigation. Routine imaging, PFT, and clinical assessments currently remain crucial for early detection and intervention in lung transplant recipients. Incorporating FEF25-75 and CT imaging in routine follow-up could help better detect allograft dysfunction at an earlier stage, allowing for timely intervention. In conclusion, this study provides valuable insights into the dynamics of dd-cfDNA in lung transplant patients developing CLAD. Although dd-cfDNA alone may not suffice as a definitive biomarker for CLAD, its incorporation in a comprehensive monitoring strategy warrants further investigation.

## Data Availability

% dd-cfDNA level dataset presented in this article is available in Supplementary Table S1 and S2. Due to ethical and privacy considerations, we cannot share raw SNP-level data derived from patient plasma samples. Requests to access the datasets should be directed to the corresponding author.

## Supplementary material

Supplementary Figure S1 Chest CT over time in RAS patients. Representative chest CTs of RAS patients. First abnormal chest CT, chest CT around preclinical blood sampling time and representative chest CT after CLAD diagnosis are included.

Supplementary Table S1 % dd-cfDNA levels in BOS patients.

Supplementary Table S2 % dd-cfDNA levels in RAS patients.
