# Supplementary figures and images for: Donor-derived cell-free DNA in chronic lung allograft dysfunction phenotypes: a pilot study
Source: Front Transplant. 2024 Dec 23;3:1513101. doi: 10.3389/frtra.2024.1513101 (PMC11701071; doi:10.3389/frtra.2024.1513101)

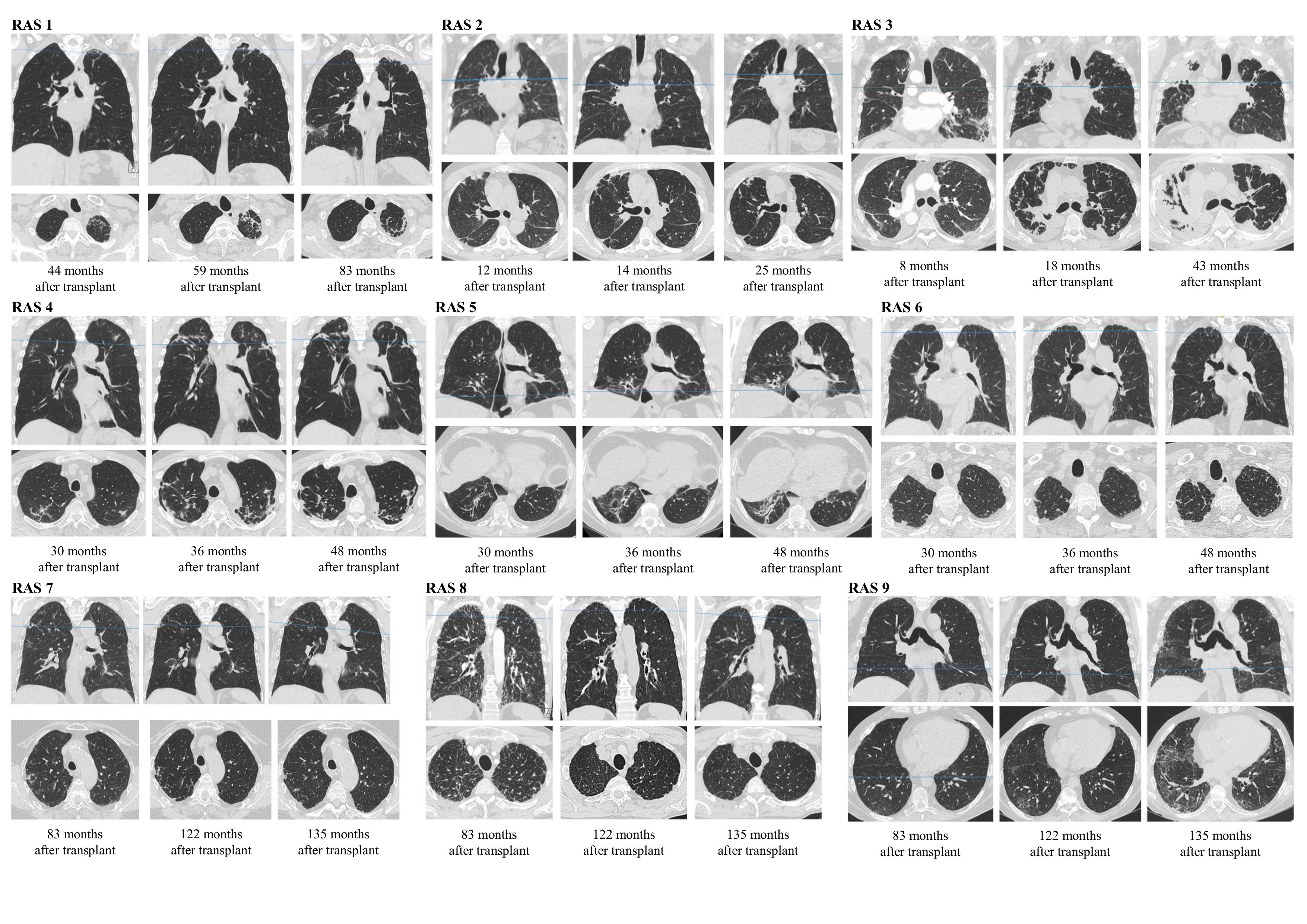

Supplement: Supplementary Figure S1 — Chest CT over time in RAS patients. Representative chest CTs of RAS patients. First abnormal chest CT, chest CT around preclinical blood sampling time and representative chest CT after CLAD diagnosis are included. [file Image1.jpeg]
