# Supplementary material for: Donor-derived cell-free DNA in chronic lung allograft dysfunction phenotypes: a pilot study
Source: Front Transplant. 2024 Dec 23;3:1513101. doi: 10.3389/frtra.2024.1513101 (PMC11701071; doi:10.3389/frtra.2024.1513101)
Supplement: Supplementary Table S1 — % dd-cfDNA levels in BOS patients. [file Table1.docx]

Supplementary Material

| **Supplementary table 1: % dd-cfDNA levels in BOS patients** | | | |
| --- | --- | --- | --- |
|  | ***Clinical condition*** | ***Postoperative day measurement*** | ***% dd-cfDNA*** |
| BOS 1 | Stable | 183 | 1,05 |
| BOS 1 | Preclinical CLAD | 735 | 1,13 |
| BOS 1 | Established CLAD | 2416 | 1,68 |
| BOS 2 | Stable | 181 | 0,16 |
| BOS 2 | Preclinical CLAD | 363 | 2,07 |
| BOS 2 | Established CLAD | 547 | 1,36 |
| BOS 3 | Stable | 97 | 0,32 |
| BOS 3 | Preclinical CLAD | 371 | 0,93 |
| BOS 3 | Established CLAD | 739 | 1,04 |
| BOS 4 | Stable | 91 | 1,23 |
| BOS 4 | Preclinical CLAD | 1102 | 2,73 |
| BOS 4 | Established CLAD | 1283 | 0,72 |
| BOS 5 | Stable | 89 | 0,11 |
| BOS 5 | Preclinical CLAD | 180 | 0,11 |
| BOS 5 | Established CLAD | 2267 | 0,27 |
| BOS 6 | Stable | 182 | 0,09 |
| BOS 6 | Preclinical CLAD | 548 | 0,19 |
| BOS 6 | Established CLAD | 818 | 0,21 |
| BOS 7 | Stable | 93 | 0,12 |
| BOS 7 | Preclinical CLAD | 363 | 0,16 |
| BOS 7 | Established CLAD | 2203 | 0,21 |
| BOS 8 | Stable | 97 | 0,66 |
| BOS 8 | Preclinical CLAD | 568 | 0,18 |
| BOS 8 | Established CLAD | 732 | 0,69 |
| BOS 9 | Stable | 372 | 1,62 |
| BOS 9 | Preclinical CLAD | 546 | 0,55 |
| BOS 9 | Established CLAD | 1099 | 0,38 |
| BOS 10 | Stable | 183 | 0,71 |
| BOS 11 | Stable | 369 | 1,32 |
