# Supplementary material for: Donor-derived cell-free DNA in chronic lung allograft dysfunction phenotypes: a pilot study
Source: Front Transplant. 2024 Dec 23;3:1513101. doi: 10.3389/frtra.2024.1513101 (PMC11701071; doi:10.3389/frtra.2024.1513101)
Supplement: Supplementary Table S2 — % dd-cfDNA levels in RAS patients. [file Table2.docx]

Supplementary Material

| **Supplementary table 2: % dd-cfDNA levels in RAS patients** | | | |
| --- | --- | --- | --- |
|  | ***Clinical condition*** | ***Postoperative day measurement*** | ***% dd-cfDNA*** |
| RAS 1 | Stable | 560 | 0,32 |
| RAS 1 | Preclinical CLAD | 1802 | 0,98 |
| RAS 1 | Established CLAD | 2502 | 5,95 |
| RAS 2 | Stable | 180 | 0,09 |
| RAS 2 | Preclinical CLAD | 369 | 2,95 |
| RAS 2 | Established CLAD | 732 | 0,27 |
| RAS 3 | Preclinical CLAD | 553 | 1,41 |
| RAS 3 | Established CLAD | 1643 | 0,96 |
| RAS 4 | Stable | 364 | 0,38 |
| RAS 4 | Preclinical CLAD | 1094 | 1,36 |
| RAS 4 | Established CLAD | 1454 | 0,14 |
| RAS 5 | Stable | 363 | 4,16 |
| RAS 5 | Preclinical CLAD | 744 | 0,7 |
| RAS 5 | Established CLAD | 2017 | 1,16 |
| RAS 6 | Stable | 415 | 3,94 |
| RAS 6 | Preclinical CLAD | 1530 | 1,58 |
| RAS 6 | Established CLAD | 2830 | 0,06 |
| RAS 7 | Stable | 552 | 0,92 |
| RAS 7 | Preclinical CLAD | 1110 | 0,15 |
| RAS 7 | Established CLAD | 2216 | 0,14 |
| RAS 8 | Stable | 1908 | 0,22 |
| RAS 8 | Preclinical CLAD | 3653 | 0,18 |
| RAS 9 | Stable | 89 | 0,18 |
| RAS 9 | Preclinical CLAD | 550 | 0,16 |
| RAS 9 | Established CLAD | 2189 | 0,12 |
